# Development of Curcumin-Loaded TiO_2_-Reinforced Chitosan Monofilaments for Biocompatible Surgical Sutures

**DOI:** 10.3390/polym17040484

**Published:** 2025-02-12

**Authors:** Fatma Demirci

**Affiliations:** Department of Polymer Materials Engineering, Faculty of Engineering and Natural Sciences, Bursa Technical University, 16310 Bursa, Türkiye; fatma.demirci@btu.edu.tr

**Keywords:** dry jet–wet spinning, chitosan, suture, drug release, antioxidant activity, bioactivity

## Abstract

Sutures provide mechanical support for wound closure after various traumas and surgical operations. Absorbable sutures are increasingly favored as they eliminate the need for secondary procedures and minimize additional damage to the wound site. In this study, chitosan sutures were produced using the dry jet–wet spinning method, achieving number 7-0 sutures (approximately 76 μm diameter) with a homogeneous surface. FTIR analysis demonstrated molecular interactions between chitosan and TiO_2_ or curcumin, confirming successful incorporation. The addition of 3% TiO_2_ increased the tensile strength of chitosan sutures by 12.32%, reaching 189.41 MPa. Morphological analysis revealed smooth surfaces free of pores and bubbles, confirming the production of high-quality sutures. Radical scavenging activity analysis showed that curcumin-loaded sutures exhibited 43% scavenging ability after 125 h, which was significantly higher compared to pure chitosan sutures. In vitro antibacterial tests demonstrated that curcumin-loaded sutures provided 98.87% bacterial inactivation against *S. aureus* within 24 h. Additionally, curcumin release analysis showed a cumulative release of 77% over 25 h. The bioactivity of the sutures was verified by hydroxyapatite layer formation after incubation in simulated body fluid, supporting their potential for tissue regeneration. These findings demonstrate that TiO_2_ reinforcement and curcumin loading significantly enhance the functional properties of chitosan sutures, making them strong candidates for biocompatible and absorbable surgical applications.

## 1. Introduction

Various medical devices can be used to hold tissues together after trauma or surgery. Although significant advances have been made in tissue adhesives and other mechanical wound closure devices such as surgical staples and skin closure tapes, surgical sutures are still the most preferred method of wound closure in both human and veterinary medicine due to their easy accessibility and applicability [1,2]. Although not all of them will provide all the features, an ideal suture provides appropriate mechanical strength, causes minimal tissue damage and reaction, reduces the risk of infection, is easy to use, and is relatively inexpensive. Sutures that provide the necessary mechanical support to the wound during the healing period are preferred in many medical applications with their stated superior features and therefore the demand for sutures increases by millions of dollars every year [3]. Sutures can be classified in various categories depending on the material used (natural or synthetic), their degradability (absorbable or non-absorbable), and their physical configuration (monofilament, multifilament, twisted, or braided) [3,4].

The most basic features expected from a suture are that it reinforces the wound closure externally and is biocompatible to support wound healing [5,6]. These requirements vary depending on the type of wound and its location. For example, while tendons take weeks to heal, epithelial tissue and muscles can heal within a few days [3,7]. Although non-absorbable sutures generally have higher durability, they can cause complications such as chronic inflammation, infection, and tissue damage [6]. On the other hand, they need to be removed from the patient with a second operation. For all these reasons, the development of absorbable sutures is seen as an important development in the field of surgical sutures [2,3]. Absorbable surgical sutures can be produced from natural polymers such as catgut [8], collagen [9], and chitin [10] fibers or synthetic polymers such as polyglycolic acid [11], poly(p-dioxanone) [12], poly(lactic-co-glycolic acid) [13], poly(trimethylene carbonate) [14], and polycaprolactone [15]. Natural origin sutures stand out due to their properties such as antibacterial, antifungal, and preventing scar formation [4,16].

The limited solubility of chitin in both aqueous and organic solutions restricts its practical applications. However, chitosan, which is derived from chitin through deacetylation, offers improved solubility and is therefore more suitable for biological applications [17]. Due to its excellent biocompatibility [18], biodegradability [19], low toxicity [20], antimicrobial [21], antioxidant [22], and anticancer [23] properties, chitosan is widely regarded as a promising polymer for biomedical and pharmaceutical applications [24]. Despite these advantages, the low mechanical strength of chitosan-based sutures restricts their direct use in surgical applications. As a result, chitosan is predominantly applied as a coating material on commercial sutures. Nevertheless, recent studies have explored the direct production of sutures from chitosan, aiming to overcome its mechanical limitations while preserving its beneficial biological properties. Montenegro and Godeiro used QiGel^®^ chitosan fibers being produced by Medovent GmbH as a suture material on rats and demonstrated the superiority of chitosan sutures by comparing their biodegradable and bacteriostatic properties with different sutures [25]. Silva et al. produced drug-loaded wet-spun chitosan sutures and investigated the morphological, biodegradation, drug release, mechanical, and cytotoxic properties of the produced sutures. It was stated that N-acetyl-D-glucosamine additive reduces mechanical performance but improves properties such as biodegradability, biocompatibility, and extended drug release of chitosan sutures, making them a good candidate for absorbable sutures [4]. Perrin et al. produced wet-spun chitosan sutures from high molecular weight fungal and shrimp chitosan and low molecular weight shrimp chitosan. They revealed the chosen chitosan and solvent effect on mechanical properties of the suture. They also examined the morphological properties and biocompatibility of the produced sutures [26]. Tan et al. prepared ultra-high molecular weight chitosan with different deacetylation degrees and molecular weights, and then produced chitosan sutures with a wet-spinning process. The effects of the deacetylation degree and molecular weight of chitosan on the morphological, mechanical, swelling degree, enzymatic degradation, and cytotoxic properties of the produced sutures were investigated [3].

As mentioned, although chitosan exhibits very good properties in terms of biomedical applications, the most important parameter limiting the industrialization of chitosan in suture applications is its low mechanical performance. To address this limitation, various additives such as nanoclay [27], carbon nanotubes [28], or inorganic nanoparticles [29] have been explored in the literature to enhance the mechanical properties of chitosan. Among these, TiO_2_ was selected as the sole additive in this study due to its well-documented ability to significantly improve both the mechanical strength and bioactivity of polymeric materials. TiO_2_ nanoparticles form covalent bonds with chitosan, leading to enhanced tensile properties without compromising biocompatibility. Additionally, TiO_2_ has demonstrated superior biocompatibility [30], bioactivity [31], and antimicrobial [30,31] potential in biomedical applications compared to other additives. While carbon nanotubes may pose cytotoxicity risks, nanoclay is primarily used as a carrier for bioactive agents rather than directly enhancing bioactivity. In contrast, TiO_2_ not only reinforces the mechanical properties of the suture but also actively contributes to its bioactive characteristics [32,33,34,35,36]. However, it should be noted that the effects of TiO_2_ can vary depending on factors such as particle size, concentration, and surface chemistry. In some cases, excessive TiO_2_ content has been reported to cause aggregation, leading to a reduction in mechanical performance, or to interfere with cellular interactions, affecting bioactivity negatively [37,38,39].

Curcumin is a phytopolyphenol obtained from the *Curcuma longa* plant [40]. This bioactive compound is a multifunctional active agent known with antibacterial [41], antifungal [42], antiviral [43], antioxidant [44], anti-inflammatory [44], anticoagulant [45], antiatherosclerotic [46], anticarcinogenic [47], and hypoglycemic [48] effects. Due to these therapeutic effects, it is preferred for use in pharmaceutical and biomedical fields.

This study demonstrates the production of chitosan sutures using the dry jet–wet spinning method, which has not been previously reported in the literature for chitosan-based surgical sutures. TiO_2_ was incorporated to enhance the mechanical strength of chitosan, addressing its limitations in suture applications. Once the desired mechanical properties were achieved, curcumin was added to improve the therapeutic potential of the sutures. The obtained sutures were characterized in terms of their morphological, physicochemical, and mechanical properties. Additionally, the effects of TiO_2_ and curcumin on radical scavenging activity, biocidal performance, and bioactivity were evaluated. The release profile of curcumin from the sutures was also examined to assess its potential for sustained therapeutic effects.

## 2. Materials and Methods

### 2.1. Materials

Chitosan (85% deacetylated) was supplied from Alfa Aesar (Karlshure, Germany) and acetic acid (99.8% purity) was purchased from Sigma-Aldrich (Steinheim, Germany). To prepare the coagulation bath, sodium hydroxide (NaOH) was acquired from Sigma-Aldrich (Prague, Czech Republic) and ethanol was obtained from Alkomed (Kocaeli, Türkiye). TiO_2_ in rutile phase used as additive material was synthesized by Duman and her team at Bursa Technical University Metallurgical Materials Engineering Laboratory. The TiO_2_ has 254.2 nm particle size, 18.9 m^2^/g surface area, and 4.22 g/cm^3^ density [49]. The other additive curcumin was obtained from Gemma (Kocaeli, Türkiye) in powder form. For radical scavenging activity tests, 2,2-Diphenyl-1-(2,4,6-trinitrophenyl)hydrazyl (DPPH) was purchased from Sigma-Aldrich (Steinheim, Germany) and methanol was obtained from Merck (Darmstadt, Germany). Reagents to prepare the simulated body fluid (SBF) solution, sodium chloride (NaCl), potassium chloride (KCl), calcium chloride dihydrate (CaCl_2_·2H_2_O), magnesium chloride hexahydrate (MgCl_2_·6H_2_O), and sodium dihydrogen phosphate monohydrate (NaH_2_PO_4_·H_2_O), were purchased from Merck (Darmstadt, Germany) and sodium bicarbonate (NaHCO_3_) was obtained from Sigma-Aldrich (Steinheim, Germany). Phosphate-buffered saline (PBS, pH 7.4) was obtained from Gündüz Kimya™ (İstanbul, Türkiye). Tween 80 was acquired from KimyaLab (İstanbul, Türkiye).

### 2.2. Production of the Sutures

The dry jet–wet spinning process was used to produce chitosan sutures. Chitosan was dissolved in an acetic acid solution, taking into account both the required acid concentration and the degree of deacetylation, as referenced in Perrin et al. [26]. Based on literature studies, a 4% chitosan solution was selected for fiber spinning and dissolved in 0.239 mol/L acetic acid by stirring overnight at room temperature [4,26,50,51,52]. TiO_2_ was then added at concentrations of 1%, 3%, and 5% by weight of chitosan, and the solution was sonicated for 15 min to ensure homogeneous dispersion before being mixed overnight. For the production of curcumin-loaded sutures, 1% curcumin (by weight of chitosan) was added to the solution and mixed for 45 min at room temperature. Throughout the preparation of the suture spinning solutions, the total solids concentration was maintained at 4 wt%. The compositions of the spinning solutions of the produced sutures are given in Table 1.

Sutures were produced using a custom-made spinning unit. The prepared solutions were transferred to a 20 mL syringe and pumped into the coagulation bath at pH 13 (1:1 mL/mL; ethanol and 10% aqueous solution of NaOH) with a flowing rate of the solution of 2.5 mL/min using a syringe pump (IPS 12, Inovenso (İstanbul, Türkiye)). The fibers entered the coagulation bath after passing a 105 mm air gap, and passed through the washing bath by the help of a 61 mm diameter perforated drum. Then, the fibers were wrapped around a 620 mm diameter wheel and kept in the washing bath for approximately 1 h until the pH reached close to 7. Subsequently, the fibers were dried overnight at room temperature by stretching. While the chitosan suture produced without additives was called CS, the TiO_2_ added sutures were named CS/1T, CS/3T, and CS/5T depending on the additive ratio they contained. The suture produced by adding curcumin was named CS/3T/C.

### 2.3. Characterizaton of the Produced Sutures

The structure of the produced sutures and the effects of the TiO_2_ and curcumin addition were observed with Fourier-transform infrared spectroscopy (FTIR). FTIR spectra of the produced sutures were recorded by a PerkinElmer Spectrum Two spectrometer (Shelton, CT, USA) in the scanning range of 400 to 4000 cm^−1^ and 16 scans were made at 4 cm^−1^.

The surface morphology of the produced sutures was observed by field emission scanning electron microscopy (FE-SEM, Carl-Zeiss/Gemini 300 (Oberkochen, Germany)) before and after incubation in SBF. Ten nm gold palladium was coated on the surface of the suture samples and coated samples were imaged with 200× and 500× magnifications.

Mechanical properties of the produced sutures were determined by uniaxial tensile tests according to ASTM D2256 standard [53] with 2 mm/min strain rate, similar to previous research [4].

In vitro swelling behavior of the produced sutures was determined by the gravimetric method. The dry weights of the samples cut from the produced sutures were weighed (W_D_) and immersed in 5 mL of phosphate-buffered saline (PBS, pH 7.4, 37 °C) solution. After a 24 h incubation period, the samples were taken from solution and the wet weights of the samples (W_W_) were determined after the fluid on the sample surface was gently blotted. The swelling degrees (W_S_) of the samples for different incubation times were calculated using Equation (1):

(W_S_) = (W_W_ − W_D_), (1)

Radical scavenging activity (RSA) of the produced sutures was observed by the DPPH method. A 1 mM DPPH solution was prepared by dissolving 3.94 mg DPPH in 100 mL methanol. An amount of 20 mg of the produced sutures was placed in dark test tubes containing 4 mL of methanol and 0.5 mL of prepared methanolic DPPH solution was added to each tube and the samples were stored in a darkroom at room temperature. The absorbance of reaction solution was measured daily with a UV–Visible spectrophotometer (Scinco-NEOSYS200 (Taipei, Taiwan)) at 517 nm and the RSA% of samples was calculated using Equation (2):

RSA (%) = ((A_DPPH_ − A_SAMPLE_)/A_DPPH_) × 100, (2)

where A_DPPH_ and A_SAMPLE_ were the absorbance of the blank and reaction solutions, respectively.

To determine curcumin loading efficiency, a known amount of CS/3T/C suture sample and curcumin was dissolved in a 10.5 mL (10:10:1) ethanol/distilled water/acetic acid solution. The absorbance of the obtained solutions was measured with a UV–Visible spectrophotometer (Scinco-NEOSYS200) at 431 nm and the loading efficiency (LE%) was calculated using Equation (3):

LE (%) = ((m_C_ × A_CS/3T/C_)/(m_CS/3T/C_ × A_C_)) × 100, (3)

where m_C_ and m_CS/3T/C_ were the weight of the curcumin and curcumin-loaded suture sample dissolved in ethanol/distilled water/acetic acid solution, and A_C_ and A_CS/3T/C_ were the absorbance of the curcumin and curcumin-loaded suture sample solutions, respectively.

For curcumin release analysis, firstly the amount of curcumin in 10 mg suture was calculated according to the value obtained from the LE result and accordingly the calibration curve was obtained by determining the total curcumin ratio in the suture. In order to determine the curcumin release, 10 mg of CS/3T/C sample was immersed in 10 mL of PBS solution containing 5% Tween 80 for 48 h at 37 °C. Since curcumin was insoluble in pure PBS solution, Tween 80 was added to the solution to dissolve free-released curcumin. Samples were taken in solution at different times and measurements were taken with a UV–Visible spectrophotometer at 431 nm. The experiment was performed in triplicate.

The in vitro bioactivity of the produced sutures (CS, CS/3T, CS/3T/C) was determined by immersion in 10 × SBF (adjusted at pH 7.4 with NaHCO_3_) which contains 10 times the calcium and phosphate ion concentrations of human plasma and being prepared according to a previous study [54]. After 3 and 14 days of incubation in 10 × SBF, the suture samples were removed from the solution, gently washed with deionized water, and dried at 37 °C. After the incubation period, the surface morphology of the sutures was observed by SEM. The Ca/P ratio of the mineralized layer of the CS/3T/C sample was analyzed using an Energy Dispersive X-ray Spectrometer (EDS, Bruker™ XFlash 6I100 (Berlin, Germany)). The chemical composition of the HA layer on the CS/3T/C suture was determined by FTIR analysis.

Biocidal efficacy performance of the produced sutures was determined under dynamic contact conditions according to ASTM E2149. The suture samples were tested against *Staphylococcus aureus* (ATCC 6538) and *Escherichia coli* (ATCC 35218). Briefly, for antibacterial activity evaluation, 2.5 mg suture samples were placed in 2 mL known concentration bacterial suspension (approximately 5 × 10^5^ CFU/mL) and shaken. For 3, 6, and 24 h contact times, 100 µL of bacterial suspensions were taken and diluted with phosphate-buffered solution. The diluted solutions were spread onto Mueller-Hilton II agar media and incubated for 24 h at 37 °C. At the end of the incubation period, viable bacterial colonies were counted and the inhibition percentage (%) was calculated using Equation (4):

Reduction rate of bacteria (%) = ((A − B)/A) × 100,(4)

where A is the number of bacteria recovered from the test sample at “0” contact time, and B is the number of bacteria recovered from test samples incubated for specific periods of time, respectively.

## 3. Results and Discussion

### 3.1. Influence of the TiO_2_ Addition on the Mechanical Properties of the Sutures

The mechanical performance of the suture is of great importance both in terms of ease of operation and keeping the wound closed until tissue regeneration after application. Despite its advantages such as biocompatibility, non-toxicity, and biodegradability, chitosan is studied in the literature as a coating on sutures rather than direct suture applications due to its low mechanical properties [1]. In recent years, studies have been carried out on the production of chitosan sutures with improved mechanical strength and functional properties [4,26]. As known in the literature, TiO_2_ nanoparticles transform into titanium cation (Ti^4+^) in an acidic environment and form covalent bonds with –OH and –NH_2_ groups of the chitosan [55]. In the first part of this study, TiO_2_ was added to the chitosan suture in order to increase its mechanical properties. TiO_2_ addition effects on the mechanical properties of the sutures are given in Table 2. The tensile strength of the CS suture was 168.63 MPa and the addition of 1 wt% and 3 wt% TiO_2_ nanoparticles to the suture structure caused an increase in both tensile strength and elongation at break. This increase could be related to the molecular interaction between the TiO_2_ nanoparticles and chitosan [55]. As seen in Table 2, the tensile strength of the CS/3T suture reached 189.41 MPa, demonstrating a 12.32% improvement compared to the unmodified CS suture. However, when the TiO_2_ content increased to 5 wt%, the tensile strength dropped to 148.83 MPa, which is lower than the CS suture. Similar trends have been observed in previous studies, where excessive TiO_2_ addition to chitosan-based films led to aggregation, reducing mechanical performance [38,39].

According to the United States Pharmacopeia (USP) standards, the minimum breaking force required for 7-0 surgical sutures is 0.785 N. The breaking strength of the CS suture was measured as 0.74 N, which is slightly below the USP requirement. However, with the addition of 1 wt% and 3 wt% TiO_2_, the breaking strength increased to 0.77 N and 0.84 N, respectively, surpassing the USP standard. This indicates that the CS/3T suture meets the mechanical strength criteria defined for 7-0 surgical sutures, making it a viable candidate for clinical applications. Conversely, the CS/5T suture exhibited a breaking strength of 0.66 N, which is lower than the USP requirement. This decrease in mechanical strength can be attributed to TiO_2_ nanoparticle agglomeration, which disrupted the polymer network and reduced overall tensile performance.

In addition to tensile strength, elongation at break plays a critical role in the mechanical behavior and functional performance of surgical sutures. Sutures need to retain a degree of flexibility to accommodate tissue swelling and contraction during the healing process. When swelling occurs, the suture should stretch to prevent excessive tension on the wound and when the swelling subsides, the suture should return to its original shape and length to maintain tissue approximation [4]. The CS/3T suture had the highest elongation at break (3.37%), indicating its improved flexibility compared to the CS (2.55%) and CS/5T (2.77%) samples. This suggests that the CS/3T suture not only meets mechanical strength requirements, but also retains an appropriate level of elasticity, making it a suitable candidate for clinical applications requiring sutures with improved adaptability to dynamic wound environments. Furthermore, these findings emphasize that chitosan-based sutures reinforced with an optimized concentration of TiO_2_ can achieve the necessary mechanical performance for surgical applications while preserving their biocompatibility and biodegradability.

Since the tensile strength of the chitosan suture was increased by approximately 12 wt% with 3 wt% addition of TiO_2_, curcumin was added to the CS/3T sample in the continuation of the study, and the following analyses were carried out on the CS, CS/3T, and CS/3T/C samples.

### 3.2. Morphological Properties of the Produced Sutures

**Figure 1:**
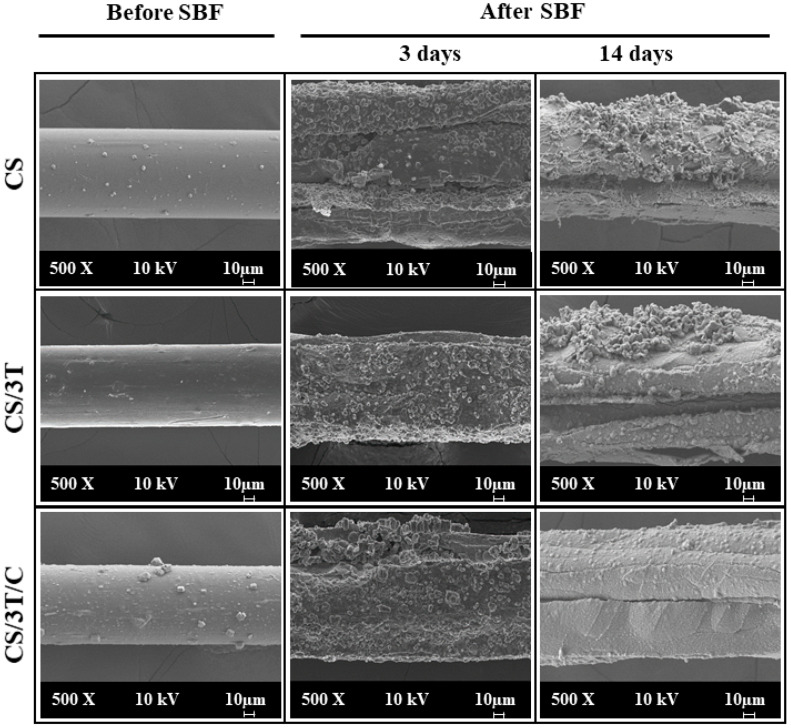
SEM images of the produced sutures before and after SBF (3 days and 14 days incubation).

The surface morphology of the produced sutures was analyzed using SEM. Figure 1 shows that both the blank and doped sutures exhibited a smooth and homogeneous surface morphology without bubbles or pores. The cross-sections of the sutures were round-shaped, indicating uniformity in fiber formation. The measured diameters of the sutures were 76.69 µm for CS, 78.15 µm for CS/3T, and 77.10 µm for CS/3T/Cur, all of which fall within the 7-0 suture range (70–99 µm) as defined by the USP. The SEM images confirm that TiO_2_ nanoparticles and curcumin were successfully incorporated into the chitosan suture structure, maintaining a uniform distribution.

In the studies in the literature, working at a high chitosan concentration such as 4 wt% and using ethanol in the coagulation bath leads to rapid and uncontrolled coagulation, often resulting in surface irregularities [26,51,56]. Additionally, coagulation in alkaline bath conditions further accelerates the phase separation process by deprotonating the charged amino groups of chitosan, allowing acetic acid and ethanol to demix more easily [26,57,58]. This rapid coagulation disrupts polymer chain organization, leading to uneven fiber morphology [26,51,56]. However, as demonstrated in Figure 1, SEM images confirm that the fiber surfaces obtained in this study are smooth and free of irregularities, despite using a high chitosan concentration (4%) and NaOH in the coagulation bath. This is likely due to the dry jet–wet spinning process, which introduces a critical air-gap stage before coagulation. Unlike wet spinning, where the polymer solution is extruded directly into the bath, the 105 mm air gap in this study allows polymer chains to partially align and stabilize under the influence of gravity before coagulation occurs. This controlled pre-orientation minimizes abrupt phase separation and prevents the coagulant from deeply penetrating the polymer matrix, leading to a more uniform and smoother fiber surface.

### 3.3. Physicochemical and Mechanical Properties of the Produced Sutures

**Figure 2:**
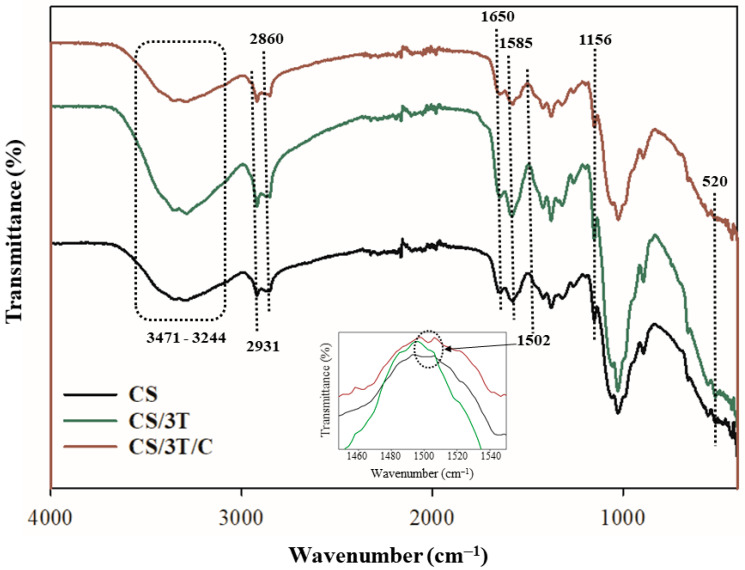
FTIR spectra of the produced suture samples.

The FTIR spectra of neat, TiO_2_, and curcumin-added sutures are given in Figure 2. The strong and broad band in the region 3471–3244 cm^−1^ corresponds to symmetric vibration of N-H and O-H. The absorption bands at 2931 and 2860 cm^−1^ can be attributed to methylene and methyl groups C-H stretching vibrations. The bands at 1650 cm^−1^ were characteristic of the C=O stretching of amide I, proving the presence of remaining N-acetyl groups in the structure of chitosan. The bands at 1585 cm^−1^ and 1156 cm^−1^ were corresponding to N-H bending of amide II and asymmetric stretching of the C-O-C bridge in the glucosamine ring, respectively. These bands overlap with the spectra of chitosan samples reported in previous studies in the literature [59,60]. It is seen that the intensities of the N-H, O-H band vibrations in the 3471–3244 cm^−1^ region and amide II band vibrations at 1585 cm^−1^ decrease with the addition of TiO_2_ and curcumin to the chitosan suture structure. These decreases were caused by the interaction between the additives and chitosan [61]. The characteristic Ti–O stretching vibration of TiO_2_ typically appears around 600 cm^−1^ in FTIR spectra [62]. However, in the CS/3T suture, this band is observed at 520 cm^−1^, indicating a slight shift. This shift suggests an interaction between TiO_2_ and the chitosan matrix, likely due to covalent or hydrogen bonding. The presence of this band confirms the successful incorporation of TiO_2_ within the suture structure. Additionally, a new vibrational band at 1502 cm^−1^ was observed exclusively in the CS/3T/C sample, which was not present in the other sutures. This band corresponds to the C=O stretching vibration of the benzene ring in the curcumin structure, confirming the successful incorporation of curcumin within the suture matrix [63].

The mechanical properties of the produced sutures are given in Table 2. The tensile strength of the CS suture was 168.63 MPa, while it was 150.90 MPa in the literature for wet-spun fibers produced at the same chitosan concentration [51]. This difference in mechanical performance is due to the production carried out with the dry jet–wet spinning process in this study. Similar results were seen in the study of Notin et al., where it was stated that physical treatment during the dry jet process improved the mechanical performance without crosslinking or post-treatment [64]. As mentioned in Section 3.1, the mechanical performance of the suture increased with the covalent bonds established between chitosan and TiO_2_ with the TiO_2_ addition. However, as shown in Table 2, the addition of curcumin led to a significant decrease in mechanical performance, with the tensile strength dropping to 128.10 MPa in the CS/3T/C sample. While the specific additive differs, a similar trend was observed in the study of Silva et al. It was observed that the tensile strength of N-Acetyl-D-Glucosamine-loaded chitosan fibers decreased by approximately 30% [4]. This decrease in mechanical performance was attributed to the decrease in secondary bond interactions caused by additive–polymer interactions [4]. This comparison is not intended to equate the two materials directly but rather to illustrate that the introduction of certain bioactive additives into a polymer matrix can disrupt intermolecular interactions, leading to reduced mechanical performance. Likewise, in this study, the weakening effect can be attributed to curcumin-induced disruptions in the polymer network, leading to reduced polymer chain interactions. Additionally, the increased swelling behavior further contributed to the mechanical deterioration. As previously mentioned, according to the USP standards, the minimum breaking force required for 7-0 surgical sutures is 0.785 N. The CS/3T/C suture exhibited the lowest breaking strength at 0.57 N, significantly below this threshold. This reduction in breaking strength correlates with the decrease in tensile strength and suggests that the mechanical stability of the CS/3T/C suture was compromised due to curcumin incorporation.

Elongation at break is another critical factor that influences the functionality of surgical sutures. Sutures must exhibit sufficient flexibility to accommodate tissue movements, particularly during swelling and contraction in the wound area. The CS/3T/C suture exhibited an elongation at break of 2.5%, indicating a slight reduction in flexibility compared to the CS suture. This decrease in elongation suggests that curcumin incorporation may have altered the polymer network, potentially reducing its ability to accommodate tissue dynamics.

On the other hand, the degree of swelling significantly affects the mechanical integrity of the suture. The swelling rates of the produced sutures after 24 h of PBS incubation were determined as 124% for CS, 99% for CS/3T, and 180% for CS/3T/C. As shown in Table 2, the addition of curcumin resulted in a decrease in tensile strength, which correlates with the significant increase in swelling behavior. In the study of Albanna et al., it was stated that increased swelling in chitosan-based sutures leads to a reduction in mechanical performance due to water absorption, which disrupts polymer chain interactions and weakens structural integrity [65]. Similarly, studies have demonstrated that curcumin incorporation into polymeric matrices can enhance swelling behavior due to its interaction with the polymer network. For instance, in the study of Ciftci et al., PVA/chitosan composite mats loaded with curcumin showed that curcumin increased the swelling capacity by affecting the polymer network structure and hydrophilicity, which in turn influenced mechanical stability [66]. Furthermore, in the study of Handy and Saeed, it was found that curcumin incorporation into chitosan-based nanocomposites altered the hydration properties of the polymer, resulting in increased swelling ratios and decreased mechanical strength. This effect was attributed to curcumin-induced structural modifications within the chitosan matrix, leading to reduced polymer chain interactions and higher water uptake [67]. Similarly, in this study, the highest swelling ratio (180%) was observed in curcumin-loaded sutures (CS/3T/C), which also exhibited the lowest tensile strength and breaking strength. This suggests that the excessive swelling behavior induced by curcumin further contributed to the mechanical deterioration of the sutures.

### 3.4. Radical Scavenging Activities of the Produced Sutures

**Figure 3:**
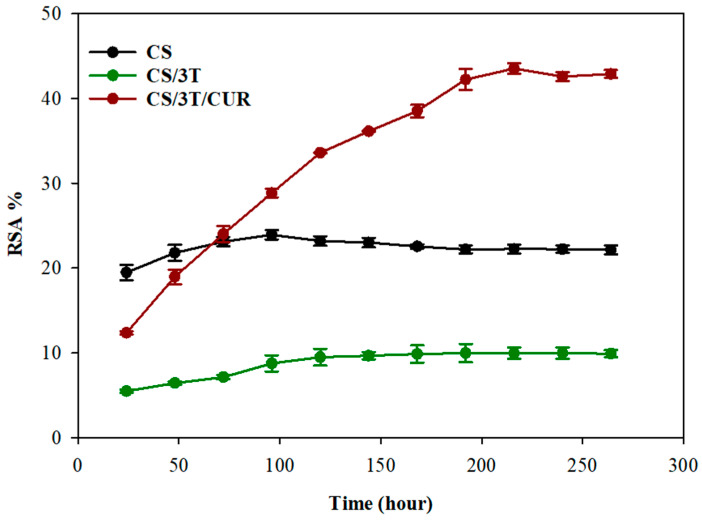
Radical scavenging activity of the produced sutures at different time intervals.

Free radicals cause many diseases and aging by damaging cells [68]. The use of antioxidant agents helps prevent the progression of diseases by capturing free radicals in human and animal cells [69,70]. To determine the antioxidant activity of the produced sutures, DPPH was used as a stable free radical that acts as a hydrogen and electron acceptor. As seen in Figure 3, the scavenging ability of CS suture was determined to be approximately 22.36%, and this value was very close to the scavenging ability determined in the study of Shanmugam et. al., who studied the same chitosan concentration (5 mg/mL) [71]. The scavenging mechanism of chitosan was attributed to an unshared electron pair of electrons of nitrogen in the C-2 position of the chitosan. In an acidic medium, this unshared electron catches the released proton and forms NH_3_^+^. Hydrogen ions of NH_3_^+^ can stabilize the free radicals causing various diseases by reacting with them [72,73]. The DPPH scavenging activity of the CS/3T was decreased to approximately 9.95%. This decrease in the DPPH scavenging activity of CS/3T is thought to be due to the binding of some –NH2 groups at the C-2 position with TiO_2_, which are responsible for the scavenging activity in chitosan. In addition to the blocking of the active groups of chitosan, the weak antioxidant activity of TiO_2_, as stated in the literature, was also effective in this decrease [74,75]. The CS/3T/CUR suture shows time-dependent activity, which confirms that the radical scavenging activity of the sutures was linked to the release of the active ingredient from the structure, similar to the literature [76,77]. As seen in the figure, while the DPPH scavenging activity of the CS/3T/CUR suture was initially between those of the CS and CS/3T sutures, it caught up with the CS suture at the 66th hour with the release of curcumin in the structure. It then continued to increase until the 125th hour and stabilized at approximately 43%.

### 3.5. In Vitro Characterization of the Produced Sutures

**Figure 4:**
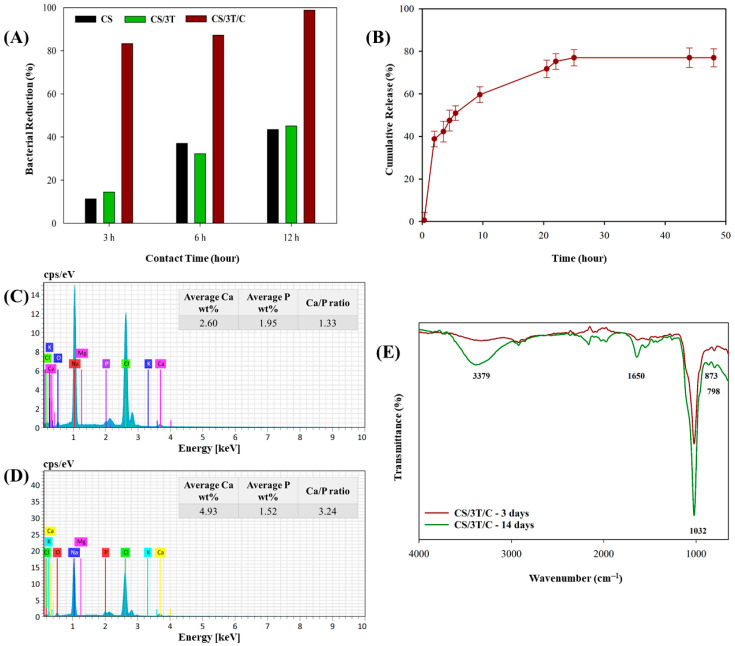
Antibacterial activity of CS, CS/3T, and CS/3T/C sutures against *S. aureus* at different time intervals (**A**), curcumin release profile of CS/3T/C suture sample (**B**), EDS spectra of the CS/3T/C suture after 3-day incubation in SBF (**C**), EDS spectra of the CS/3T/C suture after 14-day incubation in SBF (**D**), and FTIR spectra of the CS/3T/C suture after 3 and 14 days’ incubation in SBF (**E**).

Biocidal performance of the produced sutures against *S. aureus* was evaluated. The CS, CS/3T, and CS/3T/C suture swatches were challenged with 10^5^ CFU concentration *S. aureus* and the results are given in Figure 4A. Although chitosan is known as antibacterial in the literature, the amine group must be positively charged in order for chitosan to show antibacterial activity. Amine groups can only affect negatively charged groups on the bacterial cell surface when they are positively charged [78,79]. In other words, the antibacterial properties of chitosan are directly affected by the pH of the environment, and for this reason, the antibacterial activity of the suture produced only from chitosan in this study reached only 43% after 12 h. As shown in Figure 4A, the TiO_2_-doped sample showed similar antibacterial activity to CS. It is well known that TiO_2_ nanoparticles exhibit antibacterial properties mainly through a photocatalytic mechanism, where they generate reactive oxygen species (ROS), including hydroxyl radicals (–OH) and superoxide anions (O_2_^−^), under UV or visible light irradiation [38,62,80,81]. These ROS species are highly reactive and can damage bacterial membranes, proteins, and DNA, ultimately leading to cell death. However, in the absence of light, the generation of ROS is significantly reduced, limiting the antibacterial activity of TiO_2_. As the bacterial tests in this study were performed in a dark environment, TiO_2_ could not be activated to produce ROS, which explains why no significant increase in antibacterial activity was observed compared to the control (CS) sample. On the other hand, curcumin provides antibacterial activity by settling on the cell membrane of bacteria and preventing the cell from dividing and multiplying [82], and as shown in Figure 4A, the curcumin-added suture showed 83.39% effectiveness in just 3 h and provided 98.87% bacterial inactivation at the end of 24 h.

The in vitro release profile of curcumin from CS/3T/C suture was analyzed at 37 °C in PBS (at pH 7.4) solution as a simulation of body fluid, and the cumulative release against incubation time is shown in Figure 4B. The curcumin-loaded suture showed a burst release of approximately 40% in the first two hours. Although different materials were used, a similar burst release phenomenon has been reported in the literature. For example, in the study by Rezaei and Nasirpour, curcumin-loaded almond gum/PVA nanofibers exhibited a release of approximately 27% within 2 h [83]. Rezaei and Nasirpour attributed this burst release to the weaker binding of curcumin on the fiber surface to the polymer and its higher diffusion tendency. Similarly, in this study, it is hypothesized that curcumin near the surface of the high surface area suture was released more rapidly into the simulated body fluid. Although the polymer matrices are different, the general mechanism of burst release by surface-bound curcumin may be comparable. The literature suggests that burst release assists wound healing by providing immediate relief followed by a sustained release phase [84,85]. The environment in which the bioactive substance is located, along with its interaction with the surroundings, plays a crucial role in its release performance. For instance, Bui et al. demonstrated that the addition of Tween 80 to PBS significantly affects the release of curcumin from Zein nanofibers. Their study showed that both the burst release and the total release amount increased as the Tween 80 concentration increased. Specifically, while the curcumin release was only 2.5% in PBS without Tween 80 after 72 h, it reached 11.5% in PBS containing 10% Tween 80 [86]. As depicted in Figure 4B, following the initial burst release within the first two hours, the sustained release of curcumin continued for up to 25 h, ultimately reaching a total release of approximately 77%. The curcumin release profile was consistent with the antibacterial test results. After three hours, curcumin release reached approximately 40%, corresponding to an 83% bacterial inactivation rate in the CS/3T/C suture. By the 12th hour, with a curcumin release of 62%, bacterial inactivation had reached nearly 99%. It is thought that burst release will have a positive effect on the healing of the injured area by cleaning the suture area from bacteria [87].

The hydroxyapatite layer formed on the surface of a material in contact with body fluids as a result of various biological reactions provides information about the bioactivity of the material [88,89]. In in vitro applications, 10 × SBF solution is used to accelerate the formation of this layer [90]. Figure 1 (After SBF) demonstrates bonelike crystalline hydroxyapatite layer formation on produced suture samples after 3 and 14 days of incubation. Granular core apatite formation in this layer showed a cauliflower-like morphology that is characteristic for bioactive surfaces, similar to our previous study [89]. The smooth surfaces of the sutures seen in Figure 1 (Before SBF) were covered with hydroxyapatite and completely change the morphology of the sutures. In addition, an increase in suture diameters was observed due to the accumulation of hydroxyapatite on their surfaces. Similar results were seen in the study by Boccaccini et al., who coated bioactive glass on polyglactin 910 sutures. It was observed that an approximately 15 µm HA layer was formed on the surface of the sutures after 21 days of incubation in SBF solution [91]. In addition to SEM analysis, EDS was used to determine the chemical composition of the cauliflower-like clusters on the CS/3T/C suture (Figure 4C,D). The Ca/P ratio on the surface of the CS/3T/C suture increased from 1.33 after 3 days of incubation to 3.24 after 14 days in SBF. The Ca and P elements, which were found to be concentrated in the nucleation regions, confirm that the formed layer on the scaffold consists of apatite.

Figure 4E showed the FTIR spectra of the chemical composition of the apatite deposit formed on the CS/3T/C suture after 3 and 14 days of incubation in SBF. The band at 798 cm^−1^ corresponded to the P-O bond stretching, indicating the presence of the crystalline phase of the apatite structure deposited on the surface of the CS/3T/C suture [92]. While the band at 1032 cm^−1^ was associated with the triply degenerated asymmetric stretching of PO₄^3−^, the band around 873 cm^−1^ confirmed the presence of phosphate groups within the carbonated apatite structure [93,94]. On the other hand, the vibrations observed around 3379 cm^−1^ and 1650 cm^−1^, which significantly increased from day 3 to day 14, corresponded to the O–H stretching of H_2_O in the apatite layer [92,93,94].

Despite the reduced mechanical properties, the CS/3T/C suture demonstrated superior biological performance as seen in in vitro analysis. The incorporation of curcumin significantly improved antibacterial activity, bioactivity, and antioxidant performance, which are critical factors in reducing infection risks and accelerating wound healing. In surgical applications where infection control and bioactivity are prioritized over mechanical strength, such as in soft tissue repair or absorbable sutures, the benefits of curcumin-enhanced sutures may outweigh the mechanical limitations. Moreover, future studies could focus on optimizing the polymeric formulation or incorporating crosslinking strategies to enhance mechanical properties while preserving the biological advantages provided by curcumin.

## 4. Conclusions

In the present study, the dry jet–wet spinning method was successfully employed in developing smooth and homogeneous surface chitosan sutures with 7-0 suture number. The effects of the TiO_2_ and curcumin content on the physicochemical, mechanical, morphological, antioxidant, and in vitro biocidal, drug release, and bioactivity properties of the sutures were systematically investigated. In the first part of the study, it was observed that the covalent bonds established between TiO_2_ and chitosan provided an increase in the mechanical performance of chitosan sutures by up to 12.32% with 3% TiO_2_ addition. While the addition of curcumin to increase the healing performance of the suture in the surgery area provided significant improvements in this sense, it caused a decrease in mechanical performance. In conclusion, curcumin-added chitosan sutures will contribute to the healing of the surgery area due to the improved antioxidant and biocidal activity and controlled release of the drug. To my knowledge, no previous studies have investigated curcumin-loaded TiO_2_-reinforced chitosan surgical sutures. This pioneering work will pave the way for further research in this promising area and fills an important gap in the literature. By integrating innovative materials, this study not only expands the potential applications of advanced surgical sutures, but future studies can build on this foundation to improve their mechanical performance and explore broader biomedical applications.

## Figures and Tables

**Table 1:** Composition of the spinning solutions of the produced sutures.

Sample Code	Chitosan (g)	TiO_2_ (g)	Curcumin (g)
CS	0.800	0	0
CS/1T	0.792	0.008	0
CS/3T	0.776	0.024	0
CS/5T	0.760	0.040	0
CS/3T/C	0.768	0.024	0.008

**Table 2:** Mechanical properties of the produced sutures.

Sample	Breaking Strength (N)	Tensile Strength (MPa)	Elongation at Break (%)
CS	0.74 ± 0.02	168.63 ± 5.17	2.55 ± 0.18
CS/1T	0.77 ± 0.05	176.17 ± 12.23	2.63 ± 0.52
CS/3T	0.84 ± 0.04	189.41 ± 8.21	3.37 ± 0.49
CS/5T	0.66 ± 0.02	148.83 ± 5.39	2.77 ± 0.30
CS/3T/C	0.57 ± 0.01	128.10 ± 2.67	2.5 ± 0.01

## Data Availability

The original contributions presented in this study are included in the article. Further inquiries can be directed to the corresponding author.

## References

[1] Joseph B., James J., Kalarikkal N., Thomas S., Thomas S., Coates P., Whiteside B., Joseph B., Nair K. (2023). Advances in biopolymer based surgical sutures. Book Advanced Technologies and Polymer Materials for Surgical Sutures.

[2] Öksüz K.E., Kurt B., İnan Z.D.Ş., Hepokur C. (2023). Novel bioactive glass/graphene oxide-coated surgical sutures for soft tissue regeneration. ACS Omega.

[3] Tan Y., Rajoka M.S.R., Ke Z., Mehwish H.M., Deng W., Li J., Qin W., Zhao L., Wu Y. (2022). Effect of squid cartilage chitosan molecular structure on the properties of its monofilament as an absorbable surgical suture. Polymers.

[4] da Silva M.C., da Silva H.N., Alves Leal Cruz R.D.C., Sagoe Amoah S.K., de Lima Silva S.M., Lia Fook M.V. (2019). N-acetyl-D-glucosamine-loaded chitosan filaments biodegradable and biocompatible for use as absorbable surgical suture materials. Materials.

[5] Li H., Cheng F., Chávez-Madero C., Choi J., Wei X., Yi X., Zheng T., He J. (2019). Manufacturing and physical characterization of absorbable oxidized regenerated cellulose braided surgical sutures. Int. J. Biol. Macromol..

[6] Dennis C., Sethu S., Nayak S., Mohan L., Morsi Y., Manivasagam G. (2016). Suture materials—Current and emerging trends. J. Biomed. Mater. Res. Part A.

[7] Pillai C.K.S., Sharma C.P. (2010). Absorbable polymeric surgical sutures: Chemistry, production, properties, biodegradability, and performance. J. Biomater. Appl..

[8] Barbosa M.C.D.S., Silva H.N.D., Lopes D.D.S., Wanderley W.F., Rosendo R.A., Penha E.S.D., de Medeiros L.A.D.M., Silva S.M.d.L., Fook M.V.L. (2024). Biodegradable Chitosan Sutures Enhanced with N-Acetyl-D-Glucosamine: Comparative Study with Catgut Sutures. Mater. Res..

[9] Younesi M., Donmez B.O., Islam A., Akkus O. (2016). Heparinized collagen sutures for sustained delivery of PDGF-BB: Delivery profile and effects on tendon-derived cells In-Vitro. Acta Biomater..

[10] Shao K., Han B., Gao J., Jiang Z., Liu W., Liu W., Liang Y. (2016). Fabrication and feasibility study of an absorbable diacetyl chitin surgical suture for wound healing. J. Biomed. Mater. Res. Part B Appl. Biomater..

[11] Chu C.C., Williams D.F. (1983). The effect of gamma irradiation on the enzymatic degradation of polyglycolic acid absorbable sutures. J. Biomed. Mater. Res..

[12] Im J.N., Kim J.K., Kim H.K., In C.H., Lee K.Y., Park W.H. (2007). In vitro and in vivo degradation behaviors of synthetic absorbable bicomponent monofilament suture prepared with poly (p-dioxanone) and its copolymer. Polym. Degrad. Stab..

[13] Lim T.Y., Poh C.K., Wang W. (2009). Poly (lactic-co-glycolic acid) as a controlled release delivery device. J. Mater. Sci. Mater. Med..

[14] Katz A.R., Mukherjee D.P., Kaganov A.L., Gordon S. (1985). A new synthetic monofilament absorbable suture made from polytrimethylene carbonate. Surg. Gynecol. Obstet..

[15] Hu J., Song Y., Zhang C., Huang W., Chen A., He H., Zhang S., Chen Y., Tu C., Liu J. (2020). Highly aligned electrospun collagen/polycaprolactone surgical sutures with sustained release of growth factors for wound regeneration. ACS Appl. Bio Mater..

[16] Viju S., Thilagavathi G. (2013). Effect of chitosan coating on the characteristics of silk-braided sutures. J. Ind. Text..

[17] Dash M., Chiellini F., Ottenbrite R.M., Chiellini E. (2011). Chitosan—A versatile semi-synthetic polymer in biomedical applications. Prog. Polym. Sci..

[18] Rodrigues S., Dionísio M., Remunan Lopez C., Grenha A. (2012). Biocompatibility of chitosan carriers with application in drug delivery. J. Funct. Biomater..

[19] Martău G.A., Mihai M., Vodnar D.C. (2019). The use of chitosan, alginate, and pectin in the biomedical and food sector—Biocompatibility, bioadhesiveness, and biodegradability. Polymers.

[20] Rizeq B.R., Younes N.N., Rasool K., Nasrallah G.K. (2019). Synthesis, bioapplications, and toxicity evaluation of chitosan-based nanoparticles. Int. J. Mol. Sci..

[21] Tan H., Ma R., Lin C., Liu Z., Tang T. (2013). Quaternized chitosan as an antimicrobial agent: Antimicrobial activity, mechanism of action and biomedical applications in orthopedics. Int. J. Mol. Sci..

[22] Panda P.K., Sadeghi K., Park K., Seo J. (2022). Regeneration approach to enhance the antimicrobial and antioxidant activities of chitosan for biomedical applications. Polymers.

[23] Khalil K.D., Bashal A.H., Habeeb T., Abu-Dief A.M. (2024). Synergistic antibacterial and anticancer activity in gadolinium–chitosan nanocomposite films: A novel approach for biomedical applications. Appl. Organomet. Chem..

[24] Matica M.A., Aachmann F.L., Tøndervik A., Sletta H., Ostafe V. (2019). Chitosan as a wound dressing starting material: Antimicrobial properties and mode of action. Int. J. Mol. Sci..

[25] Montenegro R., Godeiro J.R.G. (2014). Chitosan based suture–focusing on the real advantages of an outstanding biomaterial. Adv. Chitin Sci..

[26] Perrin N., Mohammadkhani G., Moghadam F.H., Delattre C., Zamani A. (2022). Biocompatible fibers from fungal and shrimp chitosans for suture application. Curr. Res. Biotechnol..

[27] Farrokhi H., Koosha M., Nasirizadeh N., Salari M., Abdouss M., Li T., Gong Y. (2024). The Effect of Nanoclay Type on the Mechanical Properties and Antibacterial Activity of Chitosan/PVA Nanocomposite Films. J. Compos. Sci..

[28] Aryaei A., Jayatissa A.H., Jayasuriya A.C. (2014). Mechanical and biological properties of chitosan/carbon nanotube nanocomposite films. J. Biomed. Mater. Res. Part A.

[29] Marroquin J.B., Rhee K.Y., Park S.J. (2013). Chitosan nanocomposite films: Enhanced electrical conductivity, thermal stability, and mechanical properties. Carbohydr. Polym..

[30] Mahalakshmi S., Vijaya P. (2021). Evaluation of in-vitro biocompatibility and antimicrobial activities of titanium dioxide (TiO_2_) nanoparticles by hydrothermal method. Nano Biomed. Eng..

[31] Kiran A.S.K., Kumar T.S., Sanghavi R., Doble M., Ramakrishna S. (2018). Antibacterial and bioactive surface modifications of titanium implants by PCL/TiO_2_ nanocomposite coatings. Nanomaterials.

[32] Khan S., Garg M., Chockalingam S., Gopinath P., Kundu P.P. (2020). TiO_2_ doped chitosan/poly (vinyl alcohol) nanocomposite film with enhanced mechanical properties for application in bone tissue regeneration. Int. J. Biol. Macromol..

[33] Cano L., Pollet E., Avérous L., Tercjak A. (2017). Effect of TiO_2_ nanoparticles on the properties of thermoplastic chitosan-based nano-biocomposites obtained by mechanical kneading. Compos. Part A Appl. Sci. Manuf..

[34] Ravandi R., Heris S.Z., Hemmati S., Aghazadeh M., Davaran S., Abdyazdani N. (2024). Effects of chitosan and TiO_2_ nanoparticles on the antibacterial property and ability to self-healing of cracks and retrieve mechanical characteristics of dental composites. Heliyon.

[35] Prajapati S.K., Malaiya A., Kesharwani P., Soni D., Jain A. (2022). Biomedical applications and toxicities of carbon nanotubes. Drug Chem. Toxicol..

[36] Merino D., Tomadoni B., Salcedo M.F., Mansilla A.Y., Casalongué C.A., Alvarez V.A., Kharissova O.V., Torres-Martínez L.M., Kharisov B.I. (2022). Nanoclay as carriers of bioactive molecules applied to agriculture. Handbook of Nanomaterials and Nanocomposites for Energy and Environmental Applications.

[37] Jeon J.D., Kim M.J., Kwak S.Y. (2006). Effects of addition of TiO_2_ nanoparticles on mechanical properties and ionic conductivity of solvent-free polymer electrolytes based on porous P (VdF-HFP)/P (EO-EC) membranes. J. Power Sources.

[38] Qu L., Chen G., Dong S., Huo Y., Yin Z., Li S., Chen Y. (2019). Improved mechanical and antimicrobial properties of zein/chitosan films by adding highly dispersed nano-TiO_2_. Ind. Crops Prod..

[39] El-Wakil N.A., Hassan E.A., Abou-Zeid R.E., Dufresne A. (2015). Development of wheat gluten/nanocellulose/titanium dioxide nanocomposites for active food packaging. Carbohydr. Polym..

[40] Nocito M.C., De Luca A., Prestia F., Avena P., La Padula D., Zavaglia L., Sirianni R., Casaburi I., Puoci F., Chimento A. (2021). Antitumoral activities of curcumin and recent advances to improve its oral bioavailability. Biomedicines.

[41] Zheng D., Huang C., Huang H., Zhao Y., Khan M.R.U., Zhao H., Huang L. (2020). Antibacterial mechanism of curcumin: A review. Chem. Biodivers..

[42] Chen J., He Z.M., Wang F.L., Zhang Z.S., Liu X.Z., Zhai D.D., Chen W.D. (2016). Curcumin and its promise as an anticancer drug: An analysis of its anticancer and antifungal effects in cancer and associated complications from invasive fungal infections. Eur. J. Pharmacol..

[43] Jennings M.R., Parks R.J. (2020). Curcumin as an antiviral agent. Viruses.

[44] Menon V.P., Sudheer A.R., Aggarwal B.B., Surh Y., Shishodia S. (2007). Antioxidant and anti-inflammatory properties of curcumin. The Molecular Targets and Therapeutic Uses of Curcumin in Health and Disease, 1st ed.

[45] Kim D.C., Ku S.K., Bae J.S. (2012). Anticoagulant activities of curcumin and its derivative. BMB Rep..

[46] Riyad P., Purohit A., Karishma S., Ram H. (2022). Atherosclerotic plaque regression and HMG-CoA reductase inhibition potential of curcumin: An integrative omics and in-vivo study. J. Appl. Biol. Biotechnol.

[47] Park J., Conteas C.N. (2010). Anti-carcinogenic properties of curcumin on colorectal cancer. World J. Gastrointest. Oncol..

[48] Puneeth H.R., Sharada A.C. (2015). Antioxidant and hypoglycemic effects of curcumin pyrazole derivatives. Int. J. Pharm. Pharm. Sci..

[49] Bulut B., Duman Ş. (2021). Effects of calcination temperature on hydrothermally synthesized titanium dioxide submicron powders. Konya J. Eng. Sci..

[50] Yudin V.E., Dobrovolskaya I.P., Neelov I.M., Dresvyanina E.N., Popryadukhin P.V., Ivan’kova E.M., Elokhovskii V.Y., Kasatkin I.A., Okrugin B.M., Morganti P. (2014). Wet spinning of fibers made of chitosan and chitin nanofibrils. Carbohydr. Polym..

[51] Mohammadkhani G., Kumar Ramamoorthy S., Adolfsson K.H., Mahboubi A., Hakkarainen M., Zamani A. (2021). New solvent and coagulating agent for development of chitosan fibers by wet spinning. Polymers.

[52] Dresvyanina E.N., Dobrovol’Skaya I.P., Popryadukhin P.V., Yudin V.E., Ivan’Kova E.M., Elokhovskii V.Y., Khomenko A.Y. (2013). Influence of spinning conditions on properties of chitosan fibers. Fibre Chem..

[53] (2022). Standard Test Method for Tensile Properties of Yarns by the Single-Strand Method.

[54] Mavis B., Demirtaş T.T., Gümüşderelioğlu M., Gündüz G., Çolak Ü. (2009). Synthesis, characterization and osteoblastic activity of polycaprolactone nanofibers coated with biomimetic calcium phosphate. Acta Biomater..

[55] Hussein E.M., Desoky W.M., Hanafy M.F., Guirguis O.W. (2021). Effect of TiO_2_ nanoparticles on the structural configurations and thermal, mechanical, and optical properties of chitosan/TiO_2_ nanoparticle composites. J. Phys. Chem. Solids.

[56] Knaul J., Hooper M., Chanyi C., Creber K.A. (1998). Improvements in the drying process for wet-spun chitosan fibers. J. Appl. Polym. Sci..

[57] Nechyporchuk O., Yang Nilsson T., Ulmefors H., Köhnke T. (2020). Wet spinning of chitosan fibers: Effect of sodium dodecyl sulfate adsorption and enhanced dope temperature. ACS Appl. Polym. Mater..

[58] Pantić M., Maver U., Rožanc J., Vihar B., Andrejč D.C., Knez Ž., Novak Z. (2023). Evaluation of ethanol-induced chitosan aerogels with human osteoblast cells. Int. J. Biol. Macromol..

[59] Queiroz M.F., Teodosio Melo K.R., Sabry D.A., Sassaki G.L., Rocha H.A.O. (2014). Does the use of chitosan contribute to oxalate kidney stone formation?. Mar. Drugs.

[60] Ibekwe C.A., Oyatogun G.M., Esan T.A., Oluwasegun K.M. (2017). Synthesis and characterization of chitosan/gum arabic nanoparticles for bone regeneration. Am. J. Mater. Sci. Eng..

[61] Rachtanapun P., Klunklin W., Jantrawut P., Jantanasakulwong K., Phimolsiripol Y., Seesuriyachan P., Leksawasdi N., Chaiyaso T., Ruksiriwanich W., Phongthai S. (2021). Characterization of chitosan film incorporated with curcumin extract. Polymers.

[62] Karthikeyan K.T., Nithya A., Jothivenkatachalam K. (2017). Photocatalytic and antimicrobial activities of chitosan-TiO_2_ nanocomposite. Int. J. Biol. Macromol..

[63] Musielak E., Feliczak-Guzik A., Jaroniec M., Nowak I. (2023). Photodynamic light-triggered release of curcumin from hierarchical FAU zeolite. Catalysts.

[64] Notin L., Viton C., David L., Alcouffe P., Rochas C., Domard A. (2006). Morphology and mechanical properties of chitosan fibers obtained by gel-spinning: Influence of the dry-jet-stretching step and ageing. Acta Biomater..

[65] Albanna M.Z., Bou-Akl T.H., Walters H.L., Matthew H.W. (2012). Improving the mechanical properties of chitosan-based heart valve scaffolds using chitosan fibers. J. Mech. Behav. Biomed. Mater..

[66] Ciftci F., Özarslan A.C., Evcimen Duygulu N. (2024). Production and comprehensive characterization of PVA/chitosan transdermal composite mats loaded with bioactive curcumin; evaluation of its release kinetics, antioxidant, antimicrobial, and biocompatibility features. J. Appl. Polym. Sci..

[67] Abd El-Hady M.M., Saeed S.E.S. (2020). Antibacterial properties and pH sensitive swelling of insitu formed silver-curcumin nanocomposite based chitosan hydrogel. Polymers.

[68] Pham-Huy L.A., He H., Pham-Huy C. (2008). Free radicals, antioxidants in disease and health. Int. J. Biomed. Sci. IJBS.

[69] Yuan Y., Tan W., Zhang J., Li Q., Guo Z. (2022). Water-soluble amino functionalized chitosan: Preparation, characterization, antioxidant and antibacterial activities. Int. J. Biol. Macromol..

[70] Rasheed U., Kiani M.N., Butt M.S., Saeed H., Hanif R., Anwar S. (2024). Fabrication and biocompatibility of neem/chitosan coated silk sutures for infection control and wound healing. J. King Saud Univ. -Sci..

[71] Shanmugam A., Subhapradha N., Suman S., Ramasamy P., Saravanan R., Shanmugam V., Srinivasan A. (2012). Characterization of biopolymer “chitosan” from the shell of donacid clam *Donax scortum* (Linnaeus, 1758) and its antioxidant activity. Int. J. Pharm. Pharm. Sci.

[72] Si Trung T., Bao H.N.D. (2015). Physicochemical properties and antioxidant activity of chitin and chitosan prepared from pacific white shrimp waste. Int. J. Carbohydr. Chem..

[73] Xia W., Liu P., Zhang J., Chen J. (2011). Biological activities of chitosan and chitooligosaccharides. Food Hydrocoll..

[74] Zhang X., Liu Y., Yong H., Qin Y., Liu J., Liu J. (2019). Development of multifunctional food packaging films based on chitosan, TiO_2_ nanoparticles and anthocyanin-rich black plum peel extract. Food Hydrocoll..

[75] Ullattil S.G., Narendranath S.B., Pillai S.C., Periyat P. (2018). Black TiO_2_ nanomaterials: A review of recent advances. Chem. Eng. J..

[76] Kazan A., Demirci F. (2023). Olive leaf extract incorporated chitosan films for active food packaging. Konya J. Eng. Sci..

[77] Bi F., Qin Y., Chen D., Kan J., Liu J. (2021). Development of active packaging films based on chitosan and nano-encapsulated luteolin. Int. J. Biol. Macromol..

[78] Zhang Q., Qiao Y., Li C., Lin J., Han H., Li X., Mao J., Wang F., Wang L. (2021). Chitosan/gelatin-tannic acid decorated porous tape suture with multifunctionality for tendon healing. Carbohydr. Polym..

[79] Zhang Q., Qiao Y., Zhu J., Li Y., Li C., Lin J., Li X., Han H., Mao J., Wang F. (2021). Electroactive and antibacterial surgical sutures based on chitosan-gelatin/tannic acid/polypyrrole composite coating. Compos. Part B Eng..

[80] Zhang X., Xiao G., Wang Y., Zhao Y., Su H., Tan T. (2017). Preparation of chitosan-TiO_2_ composite film with efficient antimicrobial activities under visible light for food packaging applications. Carbohydr. Polym..

[81] Sagadevan S., Lett J.A., Vennila S., Prasath P.V., Kaliaraj G.S., Fatimah I., Léonard E., Mohammad F., Al-Lohedan H.A., Alshahateet S.F. (2021). Photocatalytic activity and antibacterial efficacy of titanium dioxide nanoparticles mediated by Myristica fragrans seed extract. Chem. Phys. Lett..

[82] Li H., Jiang Y., Yang J., Pang R., Chen Y., Mo L., Jiang Q., Qin Z. (2023). Preparation of curcumin-chitosan composite film with high antioxidant and antibacterial capacity: Improving the solubility of curcumin by encapsulation of biopolymers. Food Hydrocoll..

[83] Rezaei A., Nasirpour A. (2019). Evaluation of release kinetics and mechanisms of curcumin and curcumin-β-cyclodextrin inclusion complex incorporated in electrospun almond gum/PVA nanofibers in simulated saliva and simulated gastrointestinal conditions. BioNanoScience.

[84] Cam M.E., Yildiz S., Alenezi H., Cesur S., Ozcan G.S., Erdemir G., Edirisinghe U., Akakin D., Kuruca D.S., Kabasakal L. (2020). Evaluation of burst release and sustained release of pioglitazone-loaded fibrous mats on diabetic wound healing: An in vitro and in vivo comparison study. J. R. Soc. Interface.

[85] Huang X., Brazel C.S. (2001). On the importance and mechanisms of burst release in matrix-controlled drug delivery systems. J. Control. Release.

[86] Bui H.T., Chung O.H., Park J.S. (2014). Fabrication of electrospun antibacterial curcumin-loaded zein nanofibers. Polymer.

[87] Houshyar S., Yin H., Pope L., Zizhou R., Dekiwadia C., Hill-Yardin E.L., MC Yeung J., John S., Fox K., Tran N. (2023). Smart suture with iodine contrasting nanoparticles for computed tomography. OpenNano.

[88] Blaker J.J., Nazhat S.N., Boccaccini A.R. (2004). Development and characterisation of silver-doped bioactive glass-coated sutures for tissue engineering and wound healing applications. Biomaterials.

[89] Mutlu B., Demirci F., Duman Ş. (2024). Investigating the impact of coagulation bath temperature on the properties of biphasic calcium phosphate/poly (vinylidene fluoride)-based membrane scaffold via immersion precipitation. J. Appl. Polym. Sci..

[90] Duman Ş., Bulut B. (2021). Effect of akermanite powders on mechanical properties and bioactivity of chitosan-based scaffolds produced by 3D-bioprinting. Ceram. Int..

[91] Boccaccini A.R., Stamboulis A.G., Rashid A., Roether J.A. (2003). Composite surgical sutures with bioactive glass coating. J. Biomed. Mater. Res. Part B Appl. Biomater..

[92] Sierra L.A.Q., Escobar D.M. (2019). Characterization and bioactivity behavior of sol–gel derived bioactive vitroceramic from non-conventional precursors. Boletín De La Soc. Española De Cerámica Y Vidr..

[93] Wei D., Zhou Y., Jia D., Wang Y. (2008). Biomimetic apatite deposited on microarc oxidized anatase-based ceramic coating. Ceram. Int..

[94] Mutlu B., Demirci F., Duman Ş. (2024). Influence of boron incorporated biphasic calcium phosphate on mechanical, thermal, and biological properties of poly (vinylidene fluoride) membrane scaffold. J. Am. Ceram. Soc..

